# Use of Bed Nets and Factors That Influence Bed Net Use among Jinuo Ethnic Minority in Southern China

**DOI:** 10.1371/journal.pone.0103780

**Published:** 2014-07-31

**Authors:** Jian-wei Xu, Yuan-mei Liao, Hui Liu, Ren-hua Nie, Joshua Havumaki

**Affiliations:** 1 Yunnan Institute of Parasitic Diseases, Puer, China; 2 Health School of Ganzhou, Jiangxi Province, Ganzhou, China; 3 Foundation for Innovative New Diagnostics, Cointrin/Geneva, Switzerland; University of California, San Francisco, United States of America

## Abstract

**Background:**

Insecticide-treated nets (ITNs) are an integral part of vector control recommendations for malaria elimination in China. This study investigated the extent to which bed nets were used and which factors influence bed net use among Jinuo Ethnic Minority in China-Myanmar-Laos border areas.

**Methods and Findings:**

This study combined a quantitative household questionnaire survey and qualitative semi-structured in-depth interviews (SDI). Questionnaires were administered to 352 heads of households. SDIs were given to 20 key informants. The bed net to person ratio was 1∶2.1 (i.e., nearly one net for every two people), however only 169 (48.0%) households owned at least one net and 623 (47.2%) residents slept under bed nets the prior night. The percentages of residents who regularly slept under nets (RSUN) and slept under nets the prior night (SUNPN) were similar (48.0% vs. 47.2%, P>0.05), however the percentage correct use of nets (CUN) was significantly lower (34.5%, P<0.0001). The annual cash income per person (ACIP) was an independent factor that influenced bed net use (P<0.0001), where families with an ACIP of CNY10000 or more were much more likely to use nets. House type was strongly associated with bed net use (OR: 4.71, 95% CI: 2.81, 7.91; P<0.0001), where those with traditional wood walls and terracotta roofs were significantly more likely to use nets, and the head of household's knowledge was an independent factor (OR: 5.04, 95% CI: 2.72, 9.35; P<0.0001), where those who knew bed nets prevent malaria were significantly more likely to use nets too.

**Conclusions:**

High bed net availability does not necessarily mean higher coverage or bed net use. Household income, house type and knowledge of the ability of bed nets to prevent malaria are all independent factors that influence bed net use among Jinuo Ethnic Minority.

## Introduction

Malaria is a global disease. The World Health Organization (WHO) estimated that between 2000 and 2010, global malaria incidence decreased by 17% and malaria-specific mortality rates decreased by 26%. Reported malaria cases have reduced by more than 50% in 34 of the 99 malaria endemic countries [1]. Currently, malaria prevalence is decreasing and 32 of the 99 countries have either declared a national policy for malaria elimination or are pursuing spatially progressive elimination within their borders [2], [3]. Countries in the Asia Pacific region are making substantial progress towards eliminating malaria [4]. China has seen a great reduction in malaria burden, and is aiming to eliminate malaria by 2020 [5]. Vector control is one strategy that China is employing to eliminate malaria. This method requires that more than 90% of the population in ongoing transmission areas has at least one method of protection against mosquitoes. Therefore insecticide-treated nets (ITNs) are an integral component of the recommendations for vector control [6].

In China, Yunnan province has the heaviest burden of malaria among the country's 31 Provinces/Municipalities/Autonomous Regions, 1522 (34.0%) of 4479 malaria cases reported in the country in 2011 were from Yunnan [7]. China's border areas originally belonged to hyper-endemic malaria zones. The Jinuo People, whose language belongs to the Tibetan-Burman group of the Sino-Tibetan family [8], are an ethnic minority living in the China-Myanmar-Laos border area of the Yunnan Province in southern China. The burden of malaria is substantial amongst Jinuo People [9]. The Jinuo people used slash-and-burn agriculture for rice and maize before 1958. They then started small-scale rice cultivation in irrigated paddy fields. Rubber growing was introduced into the Jinuo Mountain Area in the 1990s. Currently, rubber typping is the main source of economic income in the region. Rubber tappers may bring malaria into the community. Workers go into the forest at 3:00–4:00 am to tap rubber. They may contract malaria in the forest and bring it into the community. The results of a survey conducted in 56 villages of Jinuo Mountain Township showed that 153 (10.3%) of 1500 residents had a malaria episode between March to November 1993. There was a 5.8% (30/513) parasite rate amongst febrile patients in Jinuo Mountain Township in 1993 [10]. This was significantly higher (x^2^ = 11.48, P = 0.0007) when compared with the 3.1% (19489/620778) rate in Yunnan province [11] in 1993. The WHO Global Malaria Program has recommended full coverage of long lasting insecticidal nets (LLINs) in areas targeted for malaria prevention [12]. However, the use of bed nets among ethnic minorities is not well researched. This study investigated the extent bed nets including untreated nets, ITNs and LLINs are used and also which factors influence bed net use among the Jinuo Ethnic Minority from July to September, the high risk season of malaria, 2011.

## Methods

### Study site and population

**Figure 1:**
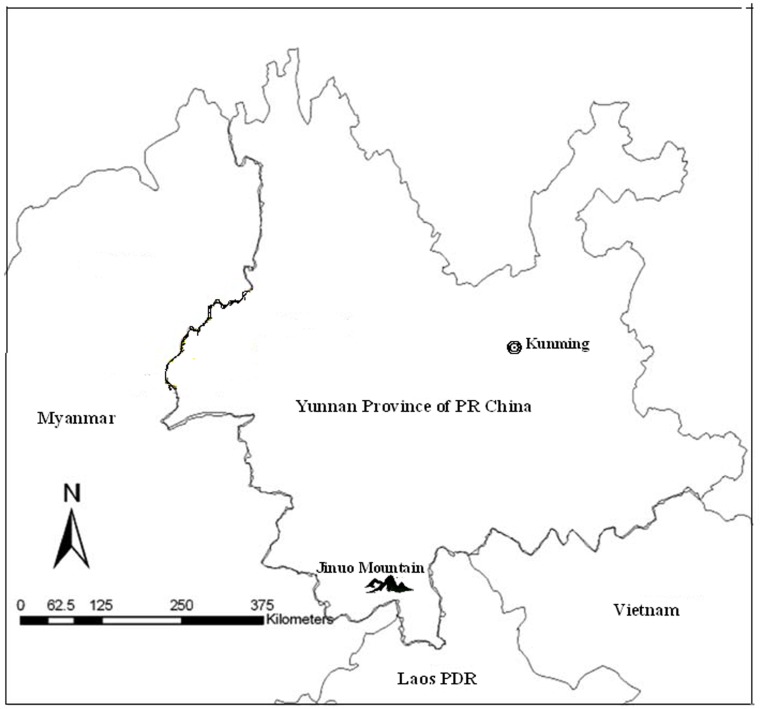
The study site: Jinuo Mountain, Yunnan Province, China.

This study was conducted on China-Myanmar-Laos border, Xishuangbanna prefecture in southern Yunnan, China (Figure 1). Jinuo tribe was recognized as an ethnic minority by the Chinese government in June 1979. It was the last of the 55 ethnic minority groups to be recognized in China. There are about 20,000 members of the Jinuo ethnic minority and they live in 56 villages on the Jinuo Mountain. The Jinuo Mountain has a tropical rain forest climate. Therefore, malaria is a substantial public health problem among the Jinuo people. Both *Plasmodium falciparum* and *P. vivax* are prevalent [7]. Malaria vectors are complex, and the primary vector *is Anopheles minimusis*. Peak malaria transmission occurs during the rainy seasons from May to November each year [13], [14].

### Household survey

The study combined a quantitative household questionnaire and qualitative semi-structured in-depth interviews (SDIs). The data collection tools (questionnaires and SDI guidelines) were developed in Chinese because the Jinuo language is only a spoken language and most heads of households understand Chinese. When a respondent did not understand Chinese, a researcher from Jinuo Township Hospital who understands both Jinuo and Chinese conducted the interview in the Jinuo language and then filled the questionnaire in Chinese. Households were the units of sampling. The list of households for sampling was obtained through the three Villager Committee Offices in Jinuo Mountain. Ten out of the 56 villages were sampled by a simple computer randomization, and then every household in each selected village was surveyed. A sample size of 350 households was required for the 5% precision around a 35% point estimate for the proportion of community residents sleeping under nets the prior night and 95% of confidence limits. A household was defined as all those eating from the same cooking pot. The interviewers first introduced the purpose of the project, the topic and type of questions. Then an oral informed consent was obtained, and finally the questionnaire was administered to heads of households. When permission was obtained, all bed nets in each household were checked to determine how much they had been used and their textile integrity.

### Semi-structured in-depth interviews

Structure in-depth Interviews (SDIs) were administered to 20 key informants to explore potential factors which influenced bed net use. The key informants consisted of two staff members that were responsible for control of communicable diseases in the Jinuo Township Hospital, seven health workers who worked for the ten selected villages, six village leaders who were appointed by the local government and five representatives of the villagers who were selected by recommendations from their fellow villagers during the household survey. The key informants discussed local health problems, malaria incidence, perception of the villagers, symptoms and treatment seeking behavior, transmission and prevention of malaria, and finally factors which influence the use of bed nets.

### Concept definitions

The use of bed nets was categorized as regularly sleeping under bed nets (RSUN), sleeping under bed nets the prior night (SUNPN) and correct use of bed nets (CUN). RSUN was defined as a person who reported habitually using nets on a daily basis. CUN was defined as an individual who closes the net and puts the hemline under the mattress before sleeping. Annual cash income per person (ACIP) was used as a surrogate for socioeconomic status because ACIP is more easily obtained. Every household owns a rubber plantation, and rubber tapping is the main source of income in the region. Nets were classified in three ways; untreated nets, ITNs and LLINs. An untreated net was either never treated or had been treated with insecticide 12 or more months prior to the survey. An ITN was treated less than 12 months prior to the survey. There is a gradual loss of insecticide over time in LLINs, reducing their protective effect. LLINs therefore expire three or more years after their production; however the dates of LLIN production could not be identified in the survey, so the LLIN were therefore defined as having been owned for three years or less. Personal characteristics of family members (PCFM) and characteristics of households and household heads (CHHH) were examined separately to determine factors that influence bed net use. PCFM are characteristics of a person in each household. CHHH are characteristics of a household and head of household who responded during the interview survey.

### Data analysis

Double data entry and cleaning of quantitative data was done in Epidata 3.1. The dataset was then analyzed in EpiInfo 7. Household ownership, coverage and use of untreated nets, ITNs and LLINs were all analyzed. Chi-squared test compared the percentage of the bed net use across different demographic groups. Sleeping under nets the prior night (SUNPN) was used as the outcome variable to decrease recall bias. Personal characteristics of family members were independent variables; a multivariate logistic model was used to assess the association of personal variables and the use of bed nets. Meanwhile, SUNPN for at least one family member was the outcome variable and the characteristics of households and their heads of households (respondents) were independent variables. The multivariate logistic analysis was conducted to analyze these difference factors and their impact on the use of nets. Qualitative data analysis was carried out to explore potential factors which influence the use of bed nets. Qualitative data analysis was conducted with support of TAMS 3.0 software. The data were encoded on the basis of emerging themes and a codebook was created. Trends in the data were identified by producing matrices which combined and compared information from the different key informants.

### Ethical approval

According to the Helsinki Declaration, ethical approval for the study was granted by the Ethics Committee of Yunnan Institute of Parasitic Diseases, China. The Ethics Committee approved a verbal consent procedure as sufficient because the study was interview-based, and did not include any human specimens. The purpose and procedures of the study were explained and disclosed to all participants before obtaining informed consent. They could choose whether or not to participate in the study, and could also refuse a response to any question at any time. The participants were for oral consent at the start of the survey and were advised that they could skip questions or end the interview at any time. Their consent was assumed if they did not refuse to answer questions. No one was coerced into participation in the study and if individuals wanted to withdraw from the study, they were allowed to do so without any issue.

## Results

### Characteristics of respondents

The total number of households was 482 across the ten sampled villages. 370 households were available at the time of sampling and 18 of them chose not to participate in the study. Three hundred and fifty two households with a total 1319 individuals participated in the questionnaire survey. The median of annual cash income per person (ACIP) of 352 households was CNY6667 (range CNY375–37500) or about US$1092 (range US$ 62–6148). Questionnaires were administered to 287 (81.5%) male and 65 (18.5%) female heads of households. The mean age of the 352 respondents was 41.3 (95% CI, 40.0, 42.5) years old. Three hundred and twenty one (91.2%) of the respondents were married. One hundred and eighty two (51.7%) of the respondents completed secondary school or higher, 154 (43.8%) had completed primary school and 16 (4.5%) were illiterate. The median number of family members was four (range 1–9) persons. The twenty key informants who were interviewed were composed of 10 males and 10 females ranging from 26 to 52 years old.

### Ownership and types of the bed nets

**Figure 2:**
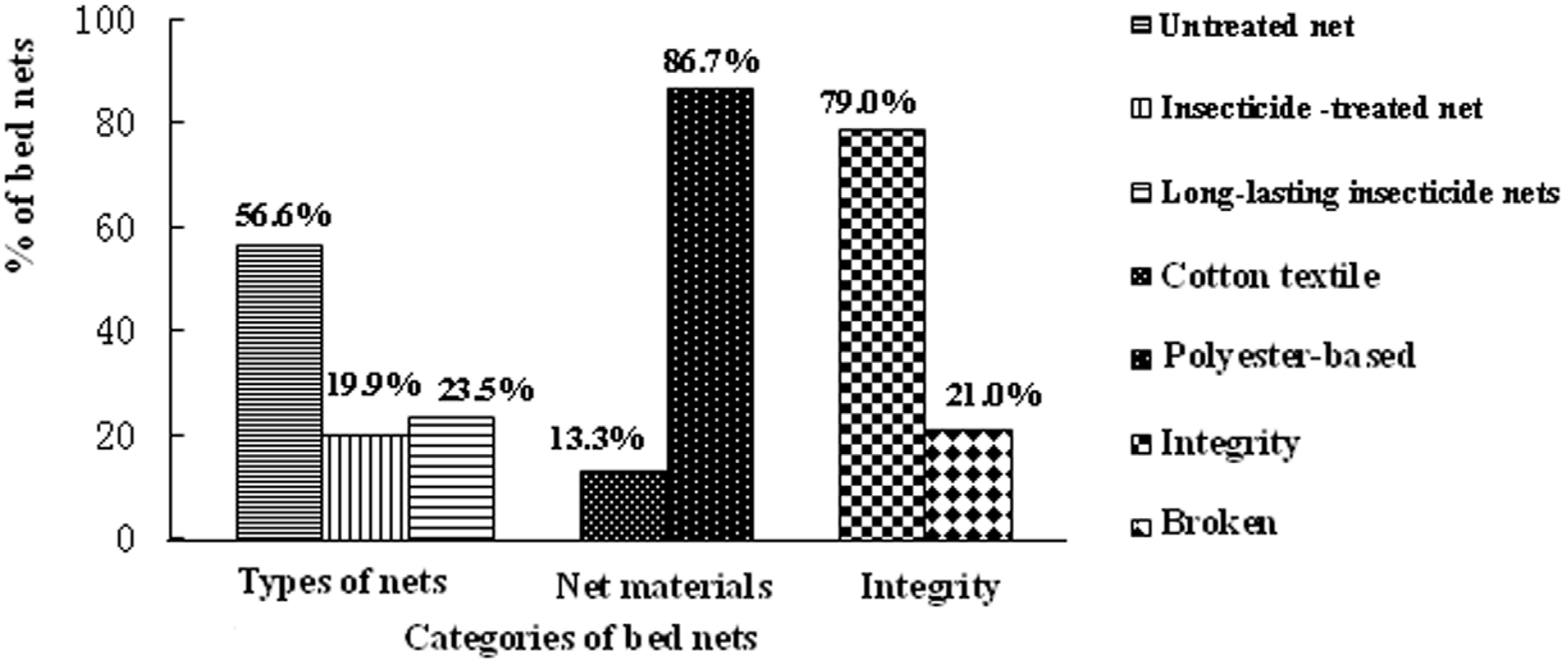
Percentage for categories of bed nets in Jinuo Ethnical Minority, Yunnan Province, China.

Ownership of bed nets was low among the Jinuo ethnic minority. One hundred and sixty nine (48.0%) households owned at least one net, reporting a total of 633 nets amongst all households. Three hundred and fifty eight (56.6%) nets were untreated nets s, 126(19.9%) were ITNs and 149 (23.5%) were LLINs. The net to person ratio was 1∶2.1(nearly one net for every two people). According to villager-self report in the survey, 482 (76.1%) of the nets were obtained by the villagers themselves commercially. Based on records of LLIN distribution in Jinuo Township Hospital, only 149 (23.5%) of free LLINs were delivered by the national malaria elimination program. One hundred and forty five (41.2%) of households owned at least one ITN or one LLIN. Eighty four (13.3%) nets were cotton textile and 549 (86.7%) were polyester-based. One hundred and thirty three (21.0%) nets had at least one hole (Figure 2).

### Use of the bed nets

The rate of bed net use was low; the key informants estimated that only half of residents consistently slept under the nets. Results from the survey revealed that 686 (52.0%) residents had never slept under nets, 633 (48.0%) residents regularly slept under the nets (RSUN), and 623(47.2%) residents slept under the nets the prior night (SUNPN). The SUNPN group included 353 (26.8%) residents who used untreated nets, 124 (9.4%) used ITNs and 146 (11.1%) used LLINs. Total RSUN and SUNPN use was similar (P>0.05), however the proportion (34.5%)of correct use of the nets (CUN) was significantly lower (P<0.0001) than RSUN and SUNPN (table 1).

**Table 1:** Bed net use among different groups of Jinuo People, Yunnan, China.

	Regular	Prior night	Correct	x^2^ value	P-value
**Sex**					
Male (n = 671)	311 (46.6%)	305 (45.5%)	224 (33.3%)	28.94	<0.0001
Female (n = 648)	322 (49.7%)	318 (49.1%)	231 (35.7%)	33.00	<0.0001
x^2^ value	1.48	1.73	0.75		
P-value	0.2244	0.1881	0.3869		
**Age**					
≤5 (n = 98)	55 (56.4%)	55 (56.4%)	41 (41.8%)	5.34	0.0455
5–14(n = 91)	42 (46.2%)	42 (46.2%)	27 (29.7%)	6.83	0.0219
15–49(n = 851)	390 (48.8%)	379 (44.5%)	300 (35.3%)	23.27	<0.0001
≥50(n = 279)	146 (52.3%)	147 (52.7%)	87 (31.2%)	34.13	<0.0001
x^2^ value	6.42	8.96	4.85		
P-value	0.0930	0.0298	0.1834		
**Educational status**					
Illiterate (n = 266)	137 (51.5%)	139 (52.3%)	90 (33.8%)	23.29	<0.0001
Primary (n = 425)	208 (48.9%)	206 (48.5%)	135 (31.8%)	33.19	<0.0001
Junior mid (n = 514)	242 (47.1%)	234 (45.5%)	190 (37.0%)	12.43	0.0010
Senior mid or higher (n = 114)	46 (40.5%)	44 (38.6%)	40 (35.1%)	0.69	0.4123
x^2^ value	4.31	6.97	2.86		
P-value	0.2303	0.0730	0.4139		
**Annual cash income per person (CNY)**					
≤6000 (n = 241)	78 (32.4%)	74 (30.7%)	49 (20.3%)	10.21	0.0027
6001–8000 (n = 453)	229 (50.6%)	228 (50.3%)	165 (36.4%)	23.91	<0.0001
8001–10000 (n = 472)	190 (40.3%)	187 (39.6%)	130 (27.5%)	21.07	<0.0001
>10000 (n = 153)	136 (88.9%)	134 (87.6%)	111 (72.6%)	17.89	0.0001
x^2^ value	138.62	139.08	130.29		
P-value	<0.0001	<0.0001	<0.0001		
**Total (n = 1319)**	**633 (48.0)**	**623 (47.2%)**	**455 (34.5%)**		**<0.0001**

Bed net use was then categorized by age, educational status and annual cash income per person (ACIP). The percentage of CUN was significantly lower than RSUN and SUNPN for all categories except for those who had completed senior middle or higher education. Rates of bed net use including RSUN, SUNPN and CUN were approximately equal between males and females (P>0.05). All five villager representatives said that “The oldest and the ill residents commonly slept under the nets more”. Additionally, children under five years old had a slightly higher rate of use than other age categories (P<0.05). It was observed that children under five years old slept under the nets more correctly than those aged 50 years or older, older people usually paid less attention to CUN and the maintenance of bed nets. Education status did not significantly predict differences in the bed net use (P>0.05). However, bed net use rates were significantly different in the four categories of economic status. Families with an ACIP of CNY10000 or more were much more likely to use nets, their rates of RSUN, SUNPN and CUN were 88.89%, 87.58% and 72.55% respectively (table 1).

### Influence factors on use of the bed nets

In summary of the qualitative study, only people of Balaxia Village considered it important to use nets; however people in nine out of the ten selected villages did not feel it was important to sleep under bed nets. Reasons include that they felt it was too hot to sleep under bed nets, they did not feel at risk for malaria infection, they could burn mosquito coils and spray insecticides instead of bed net use, they found hanging bed nets to be troublesome, and others said that they didn't have enough money to buy bed nets. The results of the multivariate logistic regression analysis (MVLRA) showed that economic status was strongly associated with bed net use. The three poorest categories were significantly less likely to use nets than those with more than CNY10000 ACIP (P<0.0001). Residents who slept outside at night were slightly less likely to use nets than those that didn't (OR: 0.58, 95% CI: 0.32, 0.94; P = 0.047) in the hot season (table 2).

**Table 2:** Personal Variables related to use of bed nets in Jinuo people, Yunnan, China.

	SUNPN*(%)	Univariate OR (95% CI)	Adjusted OR (95% CI)	P values
**Sex**				
Male (n = 671)	305 (45.5%)	0.86(0.69–1.08)	0.79 (0.54–1.99)	0.337
Female (n = 648)	318 (49.1%)	1	1	
**Age (years)**				
<15 (n = 189)	97 (51.3%)	0.95(0.64–1.39)	0.87(0.60–1.55)	0.915
15–49 (n = 851)	390 (48.8%)	0.76(0.57–1.01)	0.68(0.47–1.42)	0.169
≥50 (n = 279)	147 (52.3%)	1	1	
**Educational status**				
Illiterate & prim (n = 691)	345 (49.9)	1.26 (1.00–1.57)	1.58(0.91–1.68)	0.145
Mid or higher (n = 628)	278 (44.3)	1	1	
**Annual cash income per person (CNY)**				
≤6000 (n = 241)	74 (30.7%)	0.06 (0.03–0.11)	0.10(0.08–0.58)	<0.0001
6001–8000 (n = 453)	228 (50.3%)	0.14(0. 08–0.25)	0.18(0.12–0.59)	<0.0001
8001–10000 (n = 472)	187 (39.6%)	0.09 (0.05–0.16)	0.12 (0.07–0.46)	<0.0001
>10000 (n = 153)	134 (87.6%)	1	1	
**Rest outdoor at night of hot season**				
Yes (n = 307)	123(40.2)	0.66(0.50–0.86)	0.58(0.32–0.94)	0.047
No (n = 1012)	510(50.4)	1	1	

*SUNPN = sleeping under a net the prior night.

The qualitative information from the SDIs and household visits indicated that people living in modern houses used nets less because there are fewer holes reducing vector entry in modern houses. Additionally, they tended to use other measures such as window screens and mosquito coils. MVLRA of household variables and respondents identified that house type was strongly associated with use of the bed nets (OR: 4.71, 95% CI: 2.81, 7.91; P<0.0001), where those with traditional wood walls and terracotta roofs were significantly more likely to sleep under nets the prior night. Additionally, respondents living in houses with windows and screen doors were less likely to use nets (OR: 0.54, 95% CI: 0.29, 0.99; P = 0.045) (table 3).

**Table 3:** Household variables and respondents (Head of Household) related to use of nets in Jinuo ethnic minority, Yunnan, China.

	SUNPN1*(%)	Univariate OR (95% CI)	Adjusted OR (95% CI)	P values
**Sex of respondents**				
Male (n = 287)	142(49.5)	0.97(0.61–1.55)	0.99(0.54–1.74)	0.934
Female(n = 65)	27(41.5)	1	1	
**Age (years) of respondents**				
**<35 (n = 101)**	48(47.5)	1.38(0.80–2.38)	1.27(0.78–2.44)	0.457
**≥35 (n = 251)**	121(48.2)	1	1	
**Marriage status of respondents**				
**Married (n = 321)**	156(48.6)	1.31(0.62–2.76)	1.06 (0.47–2.88)	0.696
**Single (n = 31)**	13(41.9)	1	1	
**Family size**				
**≤5 (n = 197)**	91(46.2)	0.85(0.56–1.29)	0.88(0.54–1.36)	0.508
**>6 (n = 155)**	78(50.3)	1	1	
**Family decision**				
**Husband (n = 175)**	88(50.3)	1.12(0.79–1.82)	1.20(0.77–1.89)	0.458
**Wife or co-decision (n = 177)**	81(45.8)	1	1	
**House types**				
**Traditional (n = 173)**	118(68.2)	5.38(3.41–8.50)	4.71(2.81–7.91)	<0.0001
**Modern (n = 104)**	51(28.5)	1	1	
**Window and door screen**				
**Yes (n = 91)**	25(27.5)	0.31(0.18–0.52)	0.54(0.29–0.99)	0.045
**No (n = 261)**	144(55.2)	1	1	
**Household heads knew malaria cause**				
**Yes (n = 185)**	98(53.0)	1.53(1.00–2.32)	0.83(0.42–1.67)	0.607
**No (n = 167)**	71(42.5)	1	1	
**Household heads knew malaria transmission**				
**Yes (n = 202)**	108(53.5)	1.68(1.09–2.57)	1.32(0.64–2.69)	0.452
**No (n = 150)**	61(40.7)	1	1	
**Household heads knew bed nets against malaria infection**				
**Yes (n = 244)**	108(53.5)	4.47(2.68–7.43)	5.04(2.72–9.35)	<0.0001
**No (n = 108)**	61(40.7)	1	1	

*SUNPN1 = sleeping under a net the prior night at least one family member.

One villager head commented, “Providing information on the risk of mosquitoes such as transmission of malaria and other communicable diseases, communicating on the benefits of the bed nets, especially ITNs and LLINs and providing free bed nets will promote the use of nets”. Results of MVLRA showed that bed net use was not associated with head of household's knowledge about malaria transmission, however it was closely related to the knowledge that the bed nets prevent malaria infection (OR: 5.04, 95% CI: 2.72, 9.35; P<0.0001), where those who knew bed nets preventing malaria were significantly more likely to use nets (table 3). A village health worker in Balaxia, the site of a malaria outbreak in 2000, said, “In the malaria outbreak, many people contracted malaria. The outbreak improved awareness and knowledge of malaria among the villagers. They know bed nets are effective in preventing malaria. Currently, in our village, bed nets are available in every house and most people use them. Furthermore, bed net use has become part of our normal living habits. Most of the villagers, including those living in modern houses, always sleep under the nets”. The village head of Balaxia commented “Not only do nets prevent mosquito bites, but they also prevent nuisances caused by other pests such as cockroaches, bedbugs and fleas. Additionally, the nets are dustproof, windproof and preserve warmth in the winter”.

## Discussion

Yunnan province has one of greatest burdens of malaria in China, and Jinuo Mountain is one of the highest endemic areas in China. Of 352 households visited, 169 of them had any nets. Together these 169 houses owned 633 nets. Only 47.2% of 1319 residents surveyed had slept under them the previous night. Two hundred and fifty (19.0%) of the nets were ITNs or LLINs. One hundred and forty five (41.2%) households owned at least one LLIN or ITN. The results of a survey for the Fifth Round of China Global Fund Malaria Project in 2007 showed that 65.8% (25797/39234) of households owned at least one net and 8.68%(3404/39234) owned at least one ITN in Yunnan province [15]. With the implementation of The National Malaria Control Program, coverage and use of ITNs and LLINs increased in Yunnan. 89.7% (278/310) of households owned at least one LLIN or ITN and 30.6% (789/2582) had slept under LLINs or ITNs the previous night by 2010 [16]. This shows that the ownership of general nets, ITNs and LLINs, and the use of ITNs and LLINs among Jinuo ethnic minority are lower than the mean in the rest of Yunnan. The results of the study also indicated that Jinuo people did not have equal to access bed nets because 482 (76.1%) of the nets used were procured by villagers themselves commercially, and only 149 (23.5%) of LLINs were free from the national malaria elimination program.

More than 80% of the residents in Tanzanian households which have a net to person ratio better than 1∶4 reported using a net the previous night [17]. While a net to person ratio of 1∶2.1 among the Jinuo people only ensured that 47.2% of people slept under bed nets the previous night. MVLRA identified that annual cash income per person (ACIP) was significantly associated with the bed net use (p<0.0001). Overall, free distribution of bed nets can increase their coverage and equity. When commercial sectors are responsible for bed net availability, the ownership and use of nets is concentrated among the richest families [18]. For instance, the Wa people in a focus group discussion in Yunnan said “if nets were provided bed nets freely, we would use them” [19]. On other hand, more than other vector control methods, ITN programs largely depend on the acceptance and active involvement of communities. Involvement of communities in promoting use of bed nets also depends on their knowledge, perception, attitude and behavior towards nets [20], [21]. Knowledge, information and distribution of free bed nets are established as the first step towards encouraging the use of nets [22]. The MVLRA did not identify the association between the net use and head of household's knowledge of the cause of malaria and its transmission. However, the knowledge that bed nets prevent malaria was an independent factor for bed net use. This indicated that among the Jinuo ethnic minority, direct knowledge worked better than more technical knowledge such as the cause of malaria and the life cycle of parasites. When considering strategies to encourage behavioral changes among the Jinuo people, special attention should be given to what points should be communicated. On other hand, knowledge itself was not sufficient to guarantee bed net use. This is because knowledge does not always confer a certain behavior or action. Individual perception, awareness, the availability of nets and an environment which enables net use are necessary for behavioral change [23]–[25]. House type and availability of windows and screen doors was strongly associated with bed net use among the Jinuo Ethnical Minority. Additionally, residents who slept outdoors at night during the hot season were less likely to use nets than others. They said “sleeping under nets is uncomfortable and hanging a net is a burden” during the SDIs. The Wa people also said “sleeping under a net is too hot, so villagers prefer not to use it” [19]. These results indicate that human, socio-economic and the environmental factors can all affect the use of bed nets. Additionally, the nuisance of mosquitoes and perceived threat of malaria were the main determinants of bed net use [23]. In Balaxia village, most people have been sleeping under bed nets since the outbreak of malaria in 2000. Whereas in other nine villages, people did not sleep under bed nets because they felt like the risk of malaria was very low. The villager representatives commented, “Malaria was common ten years ago, but now it is difficult to find or has disappeared entirely”.

The epidemiological trends of malaria have changed in countries where it is targeted for elimination which are often predominantly adult men, with shared social, behavioral, and geographical risk characteristics [26]. Most Jinuo families earn their income from rubber tapping. Adults go the forest at 3:00–4:00 am to tap rubber. Therefore bed nets are unlikely to prevent malaria transmission for these rubber tappers since they are exposed at night. They might then bring malaria from the forest into the community. Bed net use within this community can prevent malaria transmission from the malaria infected workers to others. Therefore, bed net use within this community is very important for both these workers and other villagers. On the other hand, other measures, such as topical and spatial repellents for rubber tappers are needed. Additionally, among villagers, long-lasting insecticidal hammocks and mosquito traps should be used before sleeping and throughout the night for those that sleep outside [27], [28].

The study had several limitations. Firstly, considering the low prevalence of pregnant women and low malaria incidence in general, information on pregnant women was not specifically gathered. Therefore, the study has not identified whether pregnant women were more likely to sleep under nets. Secondly, if demographic groups are not similar, bias might be introduced [29]. Selection bias and information bias may have influenced the study results. One hundred and twelve (23.2%) households were not available at the time of sampling, and 18 (3.7%) households chose not to participate in the study. However it is not known why residents were away from their homes or why they opted out of participating in the study. This might have led to selection bias. However, the selection bias would be limited because 73.1% of households were involved in the study. Despite the analysis adjusting for sex and age to identify independent influence factors, a higher proportion (81.5%) of male respondents might have also caused selection bias. Data on relatively sensitive topics, such as annual cash income per person (ACIP) was obtained by self-reporting. This is a potential source information bias. In the interviews, the validity of ACIP was confirmed through the number of rubber trees owned by each household.

In Conclusion, high bed net availability does not necessarily mean higher coverage or bed net use. The household income, house type and knowledge of the ability of bed nets to prevent malaria are independent influence factors which influence bed net use among Jinuo Ethnic Minority.
